# Predictive Impact of Modified-Prognostic Nutritional Index for Acute Kidney Injury within 1-week after Living Donor Liver Transplantation

**DOI:** 10.7150/ijms.39014

**Published:** 2020-01-01

**Authors:** Ji Young Min, AMi Woo, Min Suk Chae, Sang Hyun Hong, Chul Soo Park, Jong Ho Choi, Hyun Sik Chung

**Affiliations:** 1 Department of Anesthesiology and Pain Medicine, Eunpyeong St. Mary's Hospital, College of Medicine, The Catholic University of Korea, Seoul, Republic of Korea.; 2 Department of Anesthesiology and Pain Medicine, Seoul St. Mary's Hospital, College of Medicine, The Catholic University of Korea, Seoul, Republic of Korea.

**Keywords:** Nutritional Assessment, Living donors, Liver transplantation, acute kidney injury, Prognosis.

## Abstract

**Background.** Acute kidney injury (AKI) is one of the common complications after living donor liver transplantation (LDLT) and is associated with increased mortality and morbidity. The prognostic nutritional index (PNI) has been used as a predictive model for postoperative complications. Here, we create a new predictive model based on the PNI and compared its predictive accuracy to other models in patients who underwent LDLT. **Material and Methods:** The data from 423 patients were collected retrospectively. The patients were dichotomized into the non-AKI and the AKI groups. Multivariate adjustment for significant postoperative variables based on univariate analysis was performed. A new predictive model was created using the results from logistic regression analysis, dubbed the modified-PNI model (mPNI). The area under the receiver operating characteristic curve (AUC) was generated to determine the diagnostic accuracy and cutoff value of individual models. The net reclassification improvement (NRI) and integrated discrimination improvement (IDI) were calculated to investigate diagnostic improvement by the mPNI. **Results:** Fifty-four patients (12.7 %) were diagnosed with AKI within 1-week after LDLT. The mPNI had the highest predictive accuracy (AUC = 0.823). The model of end-stage liver disease (MELD) scores and PNI were 0.793 and 0.749, respectively, and the INR and serum bilirubin were 0.705 and 0.637, respectively. The differences in the AUCs were statistically significant among the mPNI, PNI, INR, and serum bilirubin. The cutoff value for mPNI was 8.7. The NRI was 10.4% and the IDI was 3.3%. **Conclusions:** The mPNI predicted AKI within 1-week better than other scoring systems in patients who underwent LDLT. The recommended cutoff value of mPNI is 8.7.

## Introduction

Acute kidney injury (AKI) is a common complication of living donor liver transplantation (LDLT). It also is associated with prolonged length of hospital stay and high mortality (1.7 million deaths/year) 1, 2.

Patients with AKI after liver transplantation (LT) are at a risk for other morbidities, including hypertension and chronic kidney disease, even after patients recover from AKI 3. Previous research has shown that post-LT AKI was affected by hepatic ischemia-reperfusion injury and donor factors, such as high-risk grafts, resulting in higher model for end-stage liver disease (MELD) scores 4-7. The underlying mechanism for post-LT AKI is complex and different from other medical or surgical origins of AKI 4, 8. The reported incidence of post-LT AKI is between 5 to 94 percent. The wide variability is due to non-standardized AKI definitions. Furthermore, the accurate incidence, risk factors, and mortality associated with post-LT AKI remain unclear and a prognostic model is needed 8-11.

Malnutrition is associated with decreased immune system function, impaired respiratory function, and poor wound healing 12-14, as well as increased risks of postoperative morbidity and mortality and prolonged hospital stays 15, 16. Thus, nutritional status has been proven to be an independent prognostic factor and several nutritional screening tools, such as the Mini Nutritional Risk Screening and Nutritional Risk Screening, have been developed to assess nutritional risk in patients with specific diseases such as heart failure 17-19. Prognostic models have been used to predict the survival of patients using nutritional status combined with biochemical nutritional indicators, such as total cholesterol, serum albumin, and total lymphocyte count. The prognostic nutritional index (PNI) is one of the prognostic models predictive of survival 15. However, the PNI remains unexplored in patients who have undergone LDLT and it has not been used to predict AKI in post-LT patients.

In this study, we validated the clinical significance and prognostic ability of PNI and suggested a new predictive model based on PNI derived from logistic regression associated with post-LT AKI within 1-week in patients who underwent LDLT.

## Material and Methods

The patients in the study underwent LDLT between January 2011 and March 2018 at Seoul St. Mary's Hospital. Patients with chronic renal failure before LT were excluded. A total of 430 patients greater than 19 years old were enrolled in the study, retrospectively. Data were collected from the electronic medical record system. The Institutional Review Board of our hospital approved the use of the registry data.

The LDLTs were performed by the protocol for LTs of Seoul St. Mary's Hospital. A piggyback LT technique was performed. The right hepatic lobe of the donor was used for graft livers. A portocaval shunt was selectively performed in patients with minimal collateral circulation; findings on preoperative computed tomography, pressure gradient between the portal and central venous pressures of > 5 mmHg with cross-clamping the portal vein. The storage solution used for the graft livers was histidine-tryptophan-ketoglutarate (Custodiol^®^; Dr Franz-Köhler Chemie GmbH, Bensheim, Germany) solution.

Balanced anesthesia was performed using 4 - 6% desflurane or 1.5 - 2% sevoflurane with remifentanil infusion at 0.1 - 0.2 μg/kg/min. For appropriate muscle relaxation, atracurium was infused at rate of 6 - 8 μg/kg/min. Intraoperative transfusion of packed red blood cells was performed to maintain 25 - 30% hematocrit. Calcium gluconate and sodium bicarbonate were administered for serum calcium levels below 80% of the lower limit of the normal range and the serum pH below 7.15 with adequate minute ventilation.

For immunosuppression in post-LT period, our institutional regimen is consisted of tacrolimus, mycophenolate mofetil (MMF), and steroids. The level of tacrolimus was maintained at 7 to 10 ng/mL for the first postoperative month. Steroids were administered for the first postoperative month and MMF was administered until 6 months after LT. Basiliximab was administered on the day of LT and the day 4 of post-LT.

AKI was defined using the AKI network (AKIN) classification. We defined AKI who developed moderate-to-severe AKI within 1 month after LT. Moderate-to-severe AKI was defined by the AKIN classification; as peak serum creatinine (SCr) 2.0 - 2.9 times (stage II) or ≥ 3.0 times (stage III) baseline levels 20. Patients who required renal replacement therapy were also classified as having AKI. The baseline SCr was defined as the lowest creatinine within 1-month before transplantation. Patients who had a history of chronic kidney disease (CKD) before transplantation were excluded from the study.

Patients were dichotomized into two groups: the AKI and the non-AKI groups. To minimize confounding, the most recent laboratory results after LT were used in the model calculations. The PNI and the MELD score were calculated by the following formulas 15, 21, 22:

PNI = 10 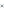
serum albumin (g/dL) + 0.005 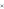 total lymphocyte count (/mm^3^)

MELD = 3.78 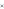
ln [serum bilirubin (mg/dL)] + 9.57 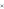 ln [serum creatinine (mg/dL)] + 11.2 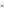 ln [international normalized ratio] + 6.43

the lower values of the PNI pointing out more severe disease and the higher values of the MELD score pointing out more severe disease.

The potentially significant postoperative 1-day variables involved in the development of AKI (*P* < 0.10) selected based on univariate analysis were analyzed by step-wise and backward logistic regression. We created a new prognostic model based on the result from logistic regression, the modified-PNI (mPNI) model, combining the PNI, conjugated serum bilirubin, and the international normalized ratio (INR).

The area under the receiver operator characteristic curve (AUC) was generated to investigate the individual diagnostic accuracies of serum bilirubin, INR, MELD score, PNI, and mPNI for AKI within 1-week after LDLT. Threshold scores, sensitivities, specificities, positive predictive values, and negative predictive values of the individual prognostic models were calculated from the results of the AUC analysis. We investigated the discrimination of individual ROC curves using the calculation of the improvement in individual AUC models by the difference in the AUCs (ΔAUC). The AUCs were compared by the method proposed by DeLong *et al.* 23.

We calculated the net reclassification improvement (NRI) and integrated discrimination improvement (IDI) to examine the incremental predictive value between the mPNI and the MELD scoring system. The NRI was calculated by the following formula by Pencina *et al.* 24.

NRI = *P*(up of event) - *P*(down of event) + *P*(down of non-event) - *P*(up of non-event)

We defined four strata of risks for AKI within 1-week after LDLT, < 5%, 5 - 15%, 15 - 20%, and > 20%.

Statistical analyses were performed by IBM SPSS Statistics for Windows, Version 19.0 (IBM Corp., Armonk, NY, USA) and MedCalc for Windows, Version 11.0 (MedCalc Software, Mariakerke, Belgium). The data of the study population are presented as the means 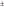 standard deviations (SD) or absolute values (proportions). Student's t-tests (for continuous variables) and Chi-squared tests (for categorical variables) were performed to compare the two groups. All statistical analyses were two-sided, and *P* value < 0.05 was considered statistically significant.

## Results

Seven cases were excluded because they were missing critical information regarding the diagnosis of AKI. Thus, a total of 423 patients were analyzed in the study. Fifty-four patients with AKI (12.7 %) were diagnosed within 1-week after LDLT. The demographics of the study population are shown in Table 1. Patients in the AKI group were younger than those in the non-AKI group. The proportion of gender showed that males were higher than females in both groups (non-AKI, 71.0%; AKI, 72.2%), however, there was no significantly different between the two groups. The AKI group has higher MELD scores and serum bilirubin levels than the non-AKI group. There were no significant differences between the two groups in recipient demographics including body mass index, cause of hepatic failure (HF), serum albumin, lymphocyte count and INR. With respect to cause of HF, viral-associated HF was a higher proportion of than non-viral HF. Among viral infections, hepatitis B virus infections were the predominant cause of HF (non-AKI, 44.2%; AKI, 44.4%), but there were no significant differences in the HF causes. With respect to donor variables, age of donors in the non-AKI group was younger than in the AKI group. Other variables were no significantly different between the two groups, including graft-to-recipient weight ratio (GWRW) and fatty changes of graft livers. The AKI group, however, showed a higher level of fatty change in the liver grafts than the non-AKI group (Table 1).

Table 2 shows the immediate postoperative laboratory data. The AKI group had statistically significant higher MELD scores, levels of serum bilirubin, and alanine transaminase (ALT) than the non-AKI group. The PNI and levels of serum total protein were significantly lower in the AKI group than the non-AKI group.

We performed multivariate adjustment of the results from univariate analysis for postoperative factors through step-wise and backward logistic regression (Table 3). We created a new predictive model based on the result from logistic regression. We named the model the modified-PNI (mPNI) model because the model included PNI, conjugated serum bilirubin levels, and INR. The new predictive model is shown below:

mPNI = 3.4 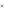 PNI - 0.7 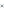 serum bilirubin (mg/dL) - 12.4 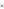 INR - 40

We compared the prediction for short-term (1-month) mortality of the mPNI, PNI, and preoperative and postoperative MELD scoring system (Table 4). All models had statistically significance for the prediction of short-term mortality. The mPNI was uniquely significant different from the PNI in resulting from the AUC analyses. The mPNI had the highest predictive accuracy, and the postoperative MELD score had the next best predictive accuracy for the prediction of short-term mortality (95%CI: 0.597 - 0.690; AUC = 0.645 and 95%CI: 0.595 - 0.689; AUC = 0.643, respectively).

Using the AUC analyses, we compared the prognostic accuracy of serum bilirubin, INR, MELD score, PNI, and mPNI for predicting AKI within 1-week after LDLT (Table 5). The mPNI had the highest predictive accuracy for post-LT AKI within 1-week of LDLT (95%CI: 0.783 - 0.858; AUC = 0.823). The MELD score had the next best predictive accuracy (95%CI: 0.753 - 0.832; AUC = 0.793). The PNI and INR had acceptable discriminative performances (95%CI: 0.705 - 0.790; AUC = 0.749 and 95%CI: 0.659 - 0.748; AUC = 0.705, respectively). However, the serum bilirubin had a poor discriminative performance (95%CI: 0.589 - 0.683; AUC = 0.637). The differences in AUCs among the individual models including the mPNI are summarized in Table 5. With the exception of the MELD score, the mPNI AUC was significantly different from those of the PNI, INR, and serum bilirubin (ΔAUC 0.074, 0.118, and 0.186;* P* = 0.006, 0.004 and <0.001, respectively). The difference between the AUCs of the mPNI and MELD score was not statistically significant (ΔAUC 0.030,* P* = 0.168). The most discriminatory cutoff values for AKI within 1-week after LDLT determined using ROC analyses were: mPNI < 8.7, PNI < 25, MELD > 23, INR > 1.9, and serum bilirubin > 5.4 (Table 5). At this cutoff point, the mPNI showed 72.2% sensitivity and 83.2% specificity. The MELD score showed 66.7% sensitivity and 79.8% specificity. The mPNI had higher sensitivity and specificity than the MELD scoring system.

Table 6 summarizes the reclassification results of individual models between the mPNI and the MELD scoring system. One hundred twenty-four individuals who were not diagnosed with AKI within 1-week after LDLT were reclassified up and the seventy-eight of individuals were reclassified down. It improved the net gain with a reclassification proportion of 0.125. Of the patients who were diagnosed with AKI within 1-week after LDLT, thirteen were reclassified up and fifteen were reclassified down. It worsened the net gain in the reclassification proportion to 0.037. Therefore, the NRI was estimated at 0.104 (95%CI: 0.034 - 0.175) and was significant difference (*P* = 0.004). The IDI was estimated at 0.033 (95%CI: 0.015 - 0.050) and was also significant (*P* < 0.001).

## Discussion

The mPNI suggested here as a new predictive model associated with nutritional status had better diagnostic accuracy than the other models tested including the MELD scoring system, for predicting AKI within 1-week after LDLT. Modification of the PNI by the addition of serum bilirubin levels and INR improved the predictive accuracy of the PNI in patients who underwent LDLT. Our results showed that the original PNI was not suitable for application to patients with hepatic diseases. The modified-nutritional index, however, had better values than the MELD score, which is made for patients with hepatic diseases and is a well-known representative scoring system for predicting the severity of hepatic diseases. Our results also showed the mPNI had better ability to predict short-term (1-month) mortality than the original PNI and MELD scoring system.

The ability to predict post-LT AKI is an important issue during the period of postoperative care, including post-transplantation. The mortality and morbidity of patients with a history of postoperative AKI are significantly higher than those of patients without such history 25. This has been proven to worsen the function of graft livers in patients with post-AKI after LT 10. Therefore, precise prediction of the probability of post-LT AKI is very important to clinicians dealing with LDLT patients, regardless of the HF cause. Previous studies have not presented prognostic models for post-LT AKI, especially based on nutritional status. However, while reported prognostic models are helpful in predicting postoperative AKI, they cannot entirely predict AKI after LDLT.

The PNI is used to determine the mortality and morbidity in patients with cardiac diseases and colorectal cancer 21, 26. However, patients with HF have not been reported the availability of the nutritional status index including the PNI. With respect to HF, the MELD scoring system has been widely and commonly used as a predictive model for outcomes and to allocate organs for transplantation in patients with end-stage liver disease. The MELD scoring system correlates well with residual liver function 27 and it also has predictive ability for postoperative AKI 7, 28. However, the MELD scoring system was not invented for predicting postoperative AKI. Therefore, a new predictive model or some modification to the original scoring for is needed to better predict post-LT AKI.

The PNI showed statistical significance between the non-AKI and the AKI groups in the present study (*P* < 0.001). However, it did not have enough predictive ability for post-LT AKI (AUC = 0.749, sensitivity 70.4%, specificity 74.0%). In general, serum bilirubin and INR play pivotal roles in enhancing the predictive ability for mortality and morbidity in patients with liver cirrhosis. In the present study, serum bilirubin showed a significant difference between the two groups (*P* = 0.015), however, the INR did not (*P* = 0.054). The AKI group showed higher serum bilirubin concentrations and longer INRs than the non-AKI group (Table 2). Therefore, the PNI is expected to be a better predictive model if it is combined with risk factors affecting the severity of hepatic function, such as serum bilirubin and INR. We considered the serum bilirubin and INR to be potentially significant factors. The serum bilirubin and INR were subjected to multivariate logistic regression to create a predictive model of post-LT AKI within 1-week after LDLT.

We evaluated the predictive ability for 1-month short-term mortality after LT of the mPNI compared to the original PNI, and pre- and post-operative MELD scoring system. All of scoring system had significant predictive ability. The best predictive model was the mPNI and the second model was the postoperative MELD scoring system (AUC = 0.645 and 0.643, respectively). However, the mPNI and PNI showed only significant difference of the predictive ability for short-term mortality among the scoring systems. Although the mPNI showed the best predictive ability for short-term mortality, the AUC showed lack of discriminatory ability to predict short-term mortality 29. Our results also showed the original PNI is not suitable to apply to predict mortality in patients with HF, and the postoperative MELD score had more predictive ability then preoperative MELD score.

We compared the AUCs of individual models, including the mPNI, PNI, MELD, INR, and serum bilirubin, to evaluate the predictive ability for post-LT AKI within 1-week after LDLT. The serum bilirubin and INR were not suitable for assessing post-LT AKI. The serum bilirubin and INR had low sensitivity, although the AUC of the INR showed an acceptable accuracy (AUC = 0.705). The serum bilirubin showed poor diagnostic accuracy and low sensitivity. The mPNI and MELD score showed good diagnostic accuracy. Of them, the mPNI had the highest predictive accuracy for AKI within 1-week after LDLT (AUC = 0.823), however, it was not significantly different from the MELD score. When the mPNI was compared to the other predictive models, all models except the MELD score showed statistical significance (*P* < 0.05).

The NRI and IDI were calculated to investigate the diagnostic improvement in the mPNI compared to the MELD score which showed the second highest predictive accuracy. The NRI and IDI confirmed that the mPNI had better predictive ability for post-LT AKI within 1-week after LDLT than the MELD scoring system (NRI 10.4%; *P* = 0.004, IDI 3.3%;* P* < 0.001).

We determined the optimal cutoff for the mPNI, PNI, MELD score, INR, and serum bilirubin with the best predictive accuracy for AKI within 1-week after LDLT using AUC analysis. Our results showed that the mPNI has a better balanced predictive ability then the other models. Using the mPNI cutoff of 8.7, the sensitivity and specificity of mPNI were as high as 72.2% and 83.2%, respectively for predicting AKI, although the AUC of mPNI was not statistically significant from the AUC of the MELD score. Thus, the mPNI can be especially useful to clinicians for predicting whether a patient undergoing LDLT will proceed to AKI after transplant (Table 5).

There are some limitations to the interpretation of the results of the present study. First, the patient's population in the present study was limited to patients who underwent LDLT. Second, the majority of causes of HF in the study patients was hepatitis B virus infection, which is common in Asia. Therefore, the progression of the disease by causes of HF, such as hepatitis B and hepatitis C virus infections may be different. Third, the calculation of PNI includes the lymphocyte count. The lymphocyte count could be affected the dose and type of administration of immunosuppressive drugs after LT. Immunosuppression regimen differs from their institution's protocol. Finally, the etiology of AKI was not adjudicated in this study, and we defined AKI who developed moderate-to-severe AKI. We exclude patient with stage 1 AKI in this study because patients with stage 2 and 3 AKI require more aggressive treatments than those with stage 1 30.

In conclusion, the mPNI has good prognostic power and is considered a useful predictive model for post-LT AKI within 1-week in patients undergoing LDLT. The diagnostic accuracy of mPNI was increased by as much as 10.4% compared to the MELD scoring system. The diagnostic superiority of mPNI over the MELD scoring system was confirmed by the results of the NRI, IDI, and AUCs. We are convinced that the suggested mPNI cutoff value of 8.7 would be helpful in providing information to transplant clinicians for predicting post-LT AKI within 1-week in patients undergoing LDLT.

## Figures and Tables

**Table 1:** Demographics of the study population.

Characteristic				Non-AKI (N=369)			AKI (N=54)			*P* value
Recipient										
	Age (years)			52	±	9	48	±	10	0.001^*^
	Gender (female/male)			107 (29.0)	/	262 (71.0)	15 (27.8)	/	39 (72.2)	0.744
	Causes of hepatic Failure									
		Hepatitis B		163		(44.2)	24		(44.4)	0.980
		Hepatitis C		81		(22.0)	13		(24.1)	
		HCC		35		(9.5)	5		(9.3)	
		Alcoholic		52		(14.1)	6		(11.1)	
		Drugs		38		(10.3)	6		(11.1)	
	BMI (kg/m^2^)			24.6	±	3.7	24.1	±	3.9	0.399
	MELD (pts)			16	±	10	22	±	13	0.003^*^
	Serum albumin (mg/dL)			3.9	±	9.0	2.9	±	0.4	0.439
	Lymphocyte (count/mm^3^)			24	±	13	21	±	12	0.122
	Serum bilirubin (total, mg/dL)			7.2	±	10.0	14.4	±	15.1	0.001^*^
	INR			3.4	±	2.2	2.2	±	1.2	0.789
Donor										
	Age (years)			35	±	12	39	±	13	0.028^*^
	Gender (female/male)			143 (38.8)	/	226 (61.2)	24 (44.4)	/	30 (55.6)	0.394
	Ischemic time (min)			105	±	61	109	±	57	0.727
	GWRW			1.20	±	0.41	1.24	±	0.39	0.551
	Fatty change of graft (%)			4.3	±	6.2	5.3	±	6.7	0.285
										

Data are presented as mean ± SD or numbers (%). calcinoma; MELD, model for end-stage liver disease. BMI, body massindex; MELD, model for end-stage liver disease; INR, international normalized ratio; GWRW, graft weight to recipient weight. *Statistically significant differences (*P* value < 0.05).

**Table 2:** Postoperative 1 day laboratory data.

	Non-AKI (N=369)			AKI (N=54)			*P* value
Serum protein (total, mg/dL)	4.5	±	0.6	4.3	±	0.6	0.010^*^
Serum albumin (mg/dL)	2.68	±	0.38	2.55	±	0.47	0.066
Lymphocyte (count/mm^3^)	6.0	±	5.2	7.9	±	7.2	0.061
PNI (pts)	27	±	3	22	±	6	<0.001^*^
Glucose (mg/dL)	228	±	96	219	±	101	0.525
INR	1.7	±	0.4	2.2	±	1.9	0.054
Serum bilirubin (total, mg/dL)	5.8	±	4.6	8.4	±	7.4	0.015^*^
MELD (pts)	19	±	6	27	±	8	<0.001^*^
ALT (mg/dL)	349	±	348	561	±	629	0.019^*^

Values are presented as mean ± standard derviation. PNI, prognostic nutritional index; MELD, model for end-stage liver disease; INR, international normalized ratio; ALT, Alanine transaminase. *Statistically significant differences (P value < 0.05).

**Table 3:** Multivariate Analysis of Predictive variables for AKI within 1-week after Liver Transplantation.

		B	S.E.	EXP (B)	95% CI			*P* value
Adjusted post-operative factors								
	PNI (pts)	0.342	0.050	1.408	1.277	-	1.552	<0.001
	Serum bilirubin (total, mg/dL)	-0.073	0.029	0.930	0.879	-	0.984	0.012
	INR	-1.241	0.413	0.289	0.129	-	0.650	0.003
	Constant	-3.998	1.390	0.018				0.004

PNI, prognostic nutritional index; INR, international normalized ratio.

**Table 4:** Comparison of the mPNI, PNI, and MELD score for short-term (1-month) survival after liver transplantation.

		Survival (N=375)			Non-Survival (N=48)			*P* value	AUC	95% CI			*P* value^a^
MELD score (pts)													
	Preoperative	17	±	10	23	±	14	0.004^*^	0.617	0.568	-	0.664	0.648
	Postoperative	20	±	6	23	±	7	<0.001^*^	0.643	0.595	-	0.689	0.968
PNI (pts)		27	±	4	25	±	5	0.029^*^	0.540	0.491	-	0.589	0.003^*^
mPNI (pts)		8.9	±	1.8	7.6	±	2.9	0.007^*^	0.645	0.597	-	0.690	Ref.

mPNI, modified-PNI; PNI, prognostic nutrional index; MELD, model for end-stage liver disease; PPV; positive predictive value, NPV; negative predictive value, AUC; area under the receiver operating characteristic curve, ΔAUC; difference in AUCs. ^a^*P* value calculated for the comparison of the mPNI vs. the other models. ^*^Statistically significant differences (P value < 0.05).

**Table 5:** Comparison of the predictive values, sensitivity, specificity, diagnostic accuracy, and difference in AUC of the mPNI, PNI, MELD, INR and Serum bilirubin for prediction of AKI within 1-week after Liver Transplantation.

Prognostic test	Threshold score	Sensitivity	Specificity	PPV	NPV	Diagnostic accuracy	ΔAUC	95%CI			* P* value^a^
mPNI (pts)	< 8.7	72.2	83.2	38.7	95.3	0.823	Ref.				
PNI (pts)	< 25	70.4	74.0	28.4	94.5	0.749	0.074	0.021	-	0.126	0.006^*^
MELD (pts)	> 23	66.7	79.8	32.6	94.2	0.793	0.030	-0.051	-	0.110	0.468
INR	> 1.9	57.4	77.5	27.2	92.5	0.705	0.118	0.037	-	0.198	0.004^*^
Serum bilirubin (total, mg/dL)	> 5.4	64.8	62.3	20.1	62.3	0.637	0.186	0.097	-	0.275	<0.001^*^

mPNI, modified-PNI; PNI, prognostic nutrional index; MELD, model for end-stage liver disease; PPV; positive predictive value, NPV; negative predictive value, AUC; area under the receiver operating characteristic curve, ΔAUC; difference in AUCs. ^a^*P* value calculated for the comparison of the mPNI vs. the other models. ^*^Statistically significant differences (P value < 0.05).

**Table 6:** Reclassification of predicted risk of AKI within 1-week after LT between the mNRI and MELD scoring system.

AKI		Model using mPNI					Reclassified		Net correctly
	Non-AKI (non-cases, N=369)	< 5%	5 - 15%	15 - 20%	≥ 20%	Total	Increased risk	Decreased risk	reclassified (%)
Model using MELD score	<5%	112 (71.8)	29 (18.6)	11 (7.1)	4 (2.6)	156			
	5%-15%	51 (50.5)	26 (25.7)	17 (16.8)	7 (6.9)	101	78	124	12.5
	15%-20%	8 (21.1)	17 (44.7)	3 (7.9)	10 (26.3)	38			
	≥ 20%	10 (13.5)	25 (33.8)	13 (17.6)	26 (35.1)	74			
								
	AKI (cases, N=54)	< 5%	5 - 15%	15 - 20%	≥ 20%	Total			
	<5%	2 (28.6)	0 (0)	1 (14.3)	4 (57.1)	7			
	5%-15%	3 (42.9)	0 (0)	3 (42.9)	1 (14.3)	7	13	15	-3.7
	15%-20%	0 (0)	1 (16.7)	1 (16.7)	4 (66.7)	6			
	≥ 20%	4 (11.8)	3 (8.8)	4 (11.8)	23 (67.6)	34			
	Net reclassification improvement		(95 % CI, P value)				0.104	(0.034 - 0.175, 0.004)	
	Integrated discrimination improvement		(95 % CI, P value)				0.033	(0.015 - 0.050, <0.001)	

AKI, acute kidkey injury; mPNI, modified-prognostic nutritional index; MELD, model for end-stage liver disease. Values are presented as numbers (%).

## Abbreviations

AK: acute kidney injury
AUC: area under the receiver operating characteristic curve
BMI: body mass index
LDLT: living donor liver transplantation
MELD: model for end-stage liver disease
PNI: prognostic nutritional index
GWRW: graft weight to recipient weight
IDI: integrated discrimination improvement
NRI: net reclassification improvement
